# High Modularity Creates Scaling Laws

**DOI:** 10.1038/s41598-018-27236-0

**Published:** 2018-06-27

**Authors:** Peter Grindrod, Desmond J. Higham

**Affiliations:** 1 Mathematical Institute, University of Oxford, England, OX2 6GG UK; 2 Department of Mathematics and Statistics, University of Strathclyde, Glasgow, G1 1XH United Kingdom

## Abstract

Scaling laws have been observed in many natural and engineered systems. Their existence can give useful information about the growth or decay of one quantitative feature in terms of another. For example, in the field of city analytics, it is has been fruitful to compare some urban attribute, such as energy usage or wealth creation, with population size. In this work, we use network science and dynamical systems perspectives to explain that the observed scaling laws, and power laws in particular, arise naturally when some feature of a complex system is measured in terms of the system size. Our analysis is based on two key assumptions that may be posed in graph theoretical terms. We assume (a) that the large interconnection network has a well-defined set of communities and (b) that the attribute under study satisfies a natural continuity-type property. We conclude that precise mechanistic laws are not required in order to explain power law effects in complex systems—very generic network-based rules can reproduce the behaviors observed in practice. We illustrate our results using Twitter interaction between accounts geolocated to the city of Bristol, UK.

## Introduction

A common theme in recent network science studies has been to partition the vertices of a given graph into *modules*, also called *clusters* or *communities*^1^, by optimizing a modularity measure^2–4^. Conceptually, graphs with high modularity have a dense set of connections between vertices within modules but only sparse connections between vertices within different modules. Thus modularity is usually employed in detecting community structure within large graphs; for example, peer-to-peer friendship and communication graphs often have a high modularity^5^. The calculation of a graph’s modularity typically involves some heuristic to search over suitable partitions so as to maximize the chosen modularity measure. Our aim is to reverse this procedure.

Suppose instead that we form a new graph by bringing together a number of separate (disjoint) graphs, each densely connected, as potential modules, and then add a few new edges between nodes from different modules so as to sparsely connect up the whole. Such a modular *graph of graphs* can form a model of network growth in various applications; see^6^ for example. Indeed, the widely used stochastic block model^7^ can be viewed as a parameter-rich extension to this idea, where edges within and between blocks of nodes are defined with different, independent, probabilities. By considering the growth of such modular graphs as further modules are added, we wish to highlight generic properties of functionals that are well defined over the space of high modularity graphs. In particular we will show that scaling laws arise for well behaved functionals, and that power laws are the dominant form of scaling laws.

We believe that this theoretical work adds clarity to the rich and growing literature on empirically observed scaling laws^8^. For example, focusing on the field of urban analytics, numerous studies have recorded the growth of some urban indicator, such as wealth, crime rate, walking speed, innovation and energy usage, in terms of population size^9–17^. Particular attention has been paid to the distinction between sublinear and superlinear scaling. The sublinear case has been associated with the efficient use of resources. Analogies have been made between urban growth and other complex systems^18–20^, and in some cases mechanistic models have been proposed in order to account for the observed behavior^9,21^.

Overall, the main contributions of this work are

- to propose a model of network growth based on sparsely connecting a collection of modules
- to impose a realistic assumption that allows us to study functionals of these graphs in terms of the functionals of their underlying modules
- to show that scaling laws arise naturally in this context
- to argue that power laws are a natural form of scaling law that, in particular, may be motivated from a dynamical systems viewpoint
- to establish that these conclusions apply generically to properties defined by local averaging over vertices, edges or other local substructures
- to show that a generalized model based on random selection of modules produces a result that is sandwiched between scaling laws
- to illustrate the relevance of these conclusions using geolocated social media data.

## Methods and Results

### Preliminaries

For any pair of sets, *S*_1_ and *S*_2_, we denote their disjoint (or discriminated) union by

This indexes the elements in the union according to which of the sets they originated in. The operation obviously extends to one defined on graphs when it is applied to the vertex sets, and consequently any and all edges in the disjoint union of the graphs are inherited directly from the component graphs. Hence the disjoint union of two graphs simply considers them both together, side by side, as a single graph, and is independent of their original indices. A matrix-level description of this operation is summarized in (8).

Let  denote the collection of finite undirected graphs with no self-loops and no repeated edges. It is obviously closed under the operation of disjoint union. In particular if *S* ∈  and $n\in {{\mathbb{Z}}}^{+}$, the positive integers, then we will write *nS* ∈  to denote the disjoint union of *n* isomorphisms of the graph *S*, that is,

Let *Q*: $\to {\mathbb{R}}$ denote a real functional defined over . Then *Q*(*S*) measures some property of *S* which relies on whatever is known about the relationships between its sub-elements (structures), or particular subsets of its sub-elements or vertices. Many graph theoretic properties, including size, average degree, edge density, averaged clustering coefficient, average centrality and various types of modularity measure can be represented by such a *Q*.

We wish to consider properties that are invariant under isomorphisms (re-indexing of the vertices) of *S*. So *a priori* the particular indexing of *S* is irrelevant and there are no distinguishable vertices. We also wish to consider those functionals *Q* that are effectively continuous with respect to the existence of edges in *S*, in the sense that if the edge set, *E*(*S*), changes by a small amount (relative to the whole) while the vertex set, *V*(*S*), remains the same, then *Q* changes by a *correspondingly small* amount.

To make this notion precise, let *S*_1_ and *S*_2_ be graphs on the same set of vertices, with nonempty *E*(*S*_1_) ⊂ *E*(*S*_2_). Then let us assume that there exists $C > 0$ such that, for all such *S*_1_ and *S*_2_ ∈ ,

1 $$|Q({S}_{2})-Q({S}_{1})|\le C\frac{|E({S}_{2}/{S}_{1})|}{|E({S}_{1})|}\mathrm{.}$$

Thus the change in *Q* is linearly bounded by the relative change in the size of the edge set. So if a new fraction *ρ* of edges are added to *S*_1_ then the change in *Q* is less than *Cρ*, independently of the choice of *S*_1_.

This condition is not satisfied in some immediately obvious cases: for example, if *Q* counts the number of disjoint connected components then a single additional edge can join two components. Yet the condition holds for properties such as the small world clustering coefficient, the vertex averaged centrality, and various types of modularity. We return to this issue later—see Theorem 1 and its accompanying discussion.

We wish to consider the consequences of condition (1) in the case where we add a few extra edges to *nS*. We add these edges between the distinct isomorphisms of *S*, so that the whole, denoted by $\widehat{nS}$, becomes simply connected. This requires approximately *n* extra connecting edges. So, under (1), for all *S* we have

$$|Q(\widehat{nS})-Q(nS)|\le C\frac{n}{E(nS)}=\frac{C}{E(S)}\mathrm{.}$$

We see that as *E*(*S*) become large the change in *Q* becomes negligible. We may (sparsely) connect up the isomorphisms of *S* without changing *Q* too much.

We emphasize that our overall aim is to study the behavior of a property *Q* as the network size grows–this captures a regime in which scaling laws are recorded in the experimental literature. Our model for network growth is to add sparse connections to the disjoint union of an increasing number of smaller graphs. This approach is motivated by empirical observations that many real networks have strong modularity. In particular^6^, showed that networks of geolocated social media interactions within UK cities can be accurately recreated from individual modules and cities of different sizes are made up of similar modules. We have now shown that if *Q* satisfies the condition (1), then the model may be simplified further; we may focus our analysis on the disjoint union. On this basis, we are now in a position to introduce and study the concept of a scaling law.

### Scaling Functions

A scaling law is a functional relationship between two physical quantities. In the urban growth context that we highlighted in the introduction, the number of nodes plays the role of population size. The network property *Q* represents a quantity of interest; for example, in^21^ this includes measures of energy consumption, economic activity, demographics, infrastructure, innovation, employment, and patterns of human behavior. Using our model of network growth, we will say that a scaling law exists if there is a *scaling function*: $G:{\mathbb{R}}\times {{\mathbb{Z}}}^{+}\to {\mathbb{R}}$ that describes how the functional *Q* behaves for disjoint unions of multiple isomorphisms of any set in  as follows:

2 $$Q(nS)=G(Q(S),n),$$

for all *S* ∈ . Applying *G* twice, first for *n* isomorphisms of *S* and then for *m* isomorphisms of *nS*, it is evident that it must satisfy the functional equation

3 $$G(Q,nm)=G(G(Q,n),m),\,G(Q,\,\mathrm{1)}=Q,$$

for all *n* and *m* in ${{\mathbb{Z}}}^{+}$ and $Q\in {{\mathbb{R}}}^{+}$. The identity (3), which is a simple, automatic consequence of a scaling law, will prove to be extremely useful in our analysis.

#### Power Law Growth and Decay

We continue by recalling the analysis in [6, Appendix A]. Based on the observation that *G*(*Q*, *n*) = *Q*/*n*^*α*^ satisfies (3), for all $\alpha \in {\mathbb{R}}$, we consider the ansatz

$$G(Q,n)=\frac{Q}{{n}^{\alpha }}+g(n),$$

where *g*(1) = 0. Suppose that *α* ≠ 0 and *g* is differentiable, and assume that (3) holds for *n* and *m* real. Then directly

$$g(nm)=\frac{g(n)}{{m}^{\alpha }}+g(m\mathrm{).}$$

Differentiating with respect to *n* and setting *n* = 1 while *g*′(1) = *αβ* for some $\beta \in {\mathbb{R}}$, we have

$$g^{\prime} (m)=\frac{\alpha \beta }{{m}^{\alpha +1}}\mathrm{.}$$

Hence, using *g*(1) = 0, we obtain $g(m)=\beta (1-\frac{1}{{m}^{\alpha }}),$ and thus

4 $$G(Q,n)=Q{n}^{-\alpha }+\beta (1-{n}^{-\alpha })$$

is a solution of all $\alpha ,\,\beta \in {\mathbb{R}}$, as derived in [6, Equation A.7].

If *α* is negative (respectively positive) then we obtain power law growth (respectively power law decay to a constant) of the property as the size of the set, measured by *n*, grows. A value of *α* different to 1 in (4) corresponds to nonlinear growth, with respect to *n*.

Now while we might hope that (3) excludes other growth possibilities, and thus explains the ubiquity of power laws observed within many applications, this is simply not so. Consider *G*(*Q*, *n*) = *Q*^*n*^. This is evidently a solution of (3). However, in the next subsection, we use a dynamical systems viewpoint in order to argue that the asymptotic power law growth identified in (4) is generic.

#### The Semigroup Property

Let us relax *n* to be real and define *H*(*Q*, *u*) = *G*(*Q*, *e*^*u*^), for *u* in ${\mathbb{R}}$. Then (3) implies

5 $$H(Q,u+v)=H(H(Q,u),v),\,\,H(Q,\,\mathrm{0)}=Q\mathrm{.}$$

So (3) is equivalent to this nonlinear semigroup property. Here *u* (and *v*) is time -like and moves *H* along the orbit of an autonomous dynamical system over ${\mathbb{R}}$ starting out from *Q* at *time* zero. So if *F*(*u*|*Q*) denotes the solution of an autonomous ordinary differential equation, with independent variable *u*, subject to the initial condition *F*(0|*Q*) = *Q*, then we may set *H*(*Q*, *u*) = *F*(*u*|*Q*) and obtain a solution of (5).

In the linear case where *F*′ = *α*(*β* − *F*) and *F*(0) = *Q*, we have *H*(*Q*, *u*) = *F*(*u*|*Q*) = *β* + (*Q* − *β*)*e*^−*uα*^, implying *G*(*Q*, *n*) = *Qn*^−*α*^ + *β*(1 − *n*^−*α*^), which agrees with the expression derived directly in (4). It is important to note that this linear case is relevant to a general nonlinear system that has approached a steady state–close to rest points any such qualitative equation is accurately described by its linearization. We therefore argue that the ubiquity of observed (near) power law growth and decay can be associated with the generic local behavior of any linear or nonlinear model that has reached equilibrium.

As an illustration, we note that power law decay applies to the usual modularity measure itself. In^6^ the authors derive a lower bound for the modularity of a disjoint union of *n* graphs, in terms of the modularity of the components, each considered in isolation (recall that 1 is always an upper bound for a graph’s modularity). Indexing the component graphs, *S*_*k*_, by *k* = 1, ..., *n*, they obtain the following lower bound on the modularity of the disjoint union, in terms of the modularities (and thus the partitions) of the separate components:

6 $$\sum _{k=1}^{n}\frac{{m}_{k}^{2}}{{m}^{2}}{R}_{k}+\sum _{k=1}^{n}\frac{{m}_{k}}{m}\frac{(m-{m}_{k})}{m}(\frac{{F}_{k}}{2{m}_{k}})\mathrm{.}$$

Here *m*_*k*_ denotes the number of edges in *S*_*k*_, *R*_*k*_ denotes the modularity of *S*_*k*_ and *F*_*k*_ denotes twice the number of edges in *S*_*k*_ that connect vertices both lying within the same module (that is, the same element of the optimal partition of *S*_*k*_ employed in defining *R*_*k*_). So *F*_*k*_/2*m*_*k*_ is simply the fraction of edges of the *k*th graph that remain within a single module.

Now let us define the measure *Q* so that *Q*(*nS*) is the lower bound given by (6) for the modularity of the disjoint union of *n* isomorphisms of any *S*, given that *Q*(*S*) is equal to *R*(*k*), the actual modularity of *S* (whence the lower bound is exact). Now applying (6) for a disjoint union of *n* isomorphisms of a graph, *S*, where *R*_*k*_ = *Q*(*S*), *m*_*k*_ = *m*/*n*, and *F*_*k*_/2*m*_*k*_ ≡ *ρ*(*S*), for all *k* = 1, ..., *n*, we may define

$$G(Q(S),n)=\frac{Q(S)}{n}+\rho (S)(1-\frac{1}{n})\mathrm{.}$$

Here *ρ*(*S*) and *Q*(*S*) are simply functional properties of the graph *S* and by construction *G*(*Q*(*S*), *n*) gives the corresponding lower bound for the modularity of *nS* that (i) is a scaling function satisfying (2), and (ii) is of the form anticipated in (4) with *α* = 1 and *β* = *ρ*.

### Constraints on the Functional *Q*(*S*)

So far, we have (a) justified a disjoint union network growth model under the assumption (1) on the property of interest and (b) argued that among the collection of scaling laws (2), a power law form is the most natural. In considering the relevance of these observations, it is natural to ask what types of functional, *Q*(*S*), admit a scaling law (2). The following result addresses this question by providing a sufficient condition.

#### **Theorem 1**

*Suppose that for all S*_0_ ∈  *there exists* $C({S}_{0}) > 0$ *such that Q satisfies*7*for all S*_1_ *and S*_2_ ∈ . *Then there exists a scaling function*, *G*, *such that* (2) *must hold for all S in* .

**Proof.** We shall assume (7) together with the converse of (2) and establish a contradiction.

If (2) fails then there must exist a pair of graphs *S*_1_ and *S*_2_ in  and a smallest integer ${n}^{\ast } > 1$, such that *Q*(*nS*_1_) = *Q*(*nS*_2_) for *n* = 1, ..., n^*^ − 1, (that is *Q* cannot distinguish between *nS*_1_ and *nS*_2_ for $n < {n}^{\ast }$) and yet *Q*(n^*^S_1_) ≠ *Q*(n^*^S_2_) (so *Q* can distinguish between n^*^S_1_ and n^*^S_2_). Such a situation would make any desired *G* ill-defined.

On the other hand if *Q*(*S*_1_) = *Q*(*S*_2_) implies *Q*(*nS*_1_) = *Q*(*nS*_2_) for all *n* ≥ 1, and for all *S*_1_ and *S*_2_ in , then *G* may be constructed pointwise, at say ($\hat{Q}$, *n*), for all values $\hat{Q}$ within the range of *Q* (over ) and all *n* (by selecting any *S* for which *Q*(*S*) = $\hat{Q}$ and assigning the corresponding value *Q*(*nS*) to *G*($\hat{Q}$*n*)).

Now, assume that *Q*(*nS*_1_) = *Q*(*nS*_2_) for *n* = 1, …, n^*^ − 1 then

Both terms on the right hand side vanish, so *Q*(n^*^S_1_) = *Q*(n^*^S_2_), giving the required contradiction.

A straightforward corollary to Theorem 1 is that any graph property defined by averaging or summing over some local property belonging to vertices, edges, or other well defined substructures, must have a scaling function. Moreover it must be linear in *Q*. To see this, let *μ*(*S*) denote the total mass in *S*, over which such an average of some local property is taken. Then

Applying this for *i* = 1, 2 we have

By Theorem 1 the “total sum” property, $\tilde{Q}$(*S*) = *μ*(*S*)*Q*(*S*), has a scaling function, $\tilde{G}$ say, and

$$\mu (nS)Q(nS)=\tilde{G}(\mu (S)Q(S),n\mathrm{).}$$

Hence

$$Q(nS)=\frac{\tilde{G}(\mu (S)Q(S),n)}{\mu (S)n}\mathrm{.}$$

But this last must hold for all *Q*(*S*) and all *μ*(*S*), which might vary independently. Yet the left-hand side is independent of *μ*, so $\tilde{G}$ must be linear in its first argument, say $\tilde{G}$(*μ*(*S*)*Q*(*S*), *n*) = *h*(*n*)*μ*(*S*)*Q*(*S*), for some function *h*(*n*), and thus

$$Q(nS)=\frac{h(n)Q(S)}{n}\equiv G(Q(S),n),$$

which is linear in *Q*, as we claimed.

### Matrix Function Viewpoint

Suppose that *Q*(*S*) = *q*(*f*(*M*)) is in the form of a real-valued functional, *q*, of a matrix valued function, *f*, of either the adjacency matrix, the graph Laplacian matrix, or the normalised graph Laplacian matrix^22^. We use *M* to denote any such matrix. If *M*_*i*_ is the corresponding matrix for each *S*_*i*_, *i* = 0, 1, 2, then that for  is block diagonal:8Hence if the *f*(*M*_*i*_) are well defined (the spectra of the *M*_*i*_ avoids any singularities and branch cuts for *f*), then *f* is also well defined for the discrete union, andThenNow suppose that this last expression is bounded above by

$$C(f({M}_{0}))|q(f({M}_{1})-q(f({M}_{2})|=C(f({M}_{0}))|Q({S}_{1})-Q({S}_{2})|,$$

for some positive functional *C*(*f*(*M*_0_)). Then *Q* has a well defined scaling function *G*, such that (2) must hold.

Simple examples occur when *q*(*f*(*M*)) denotes the sum or the modulus of product of the eigenvalues of *f*(*M*) (taking *C*(*M*_0_) to be 1 and |det(*M*_0_)| respectively). Now considering the generic properties of matrix valued functions^23^ we see that (if we consider *f* as subordinate to a complex function) then these last are simply

$$Q(S)=q(f(M))=\sum _{i=1}^{n}f({\lambda }_{i})\,\,{\rm{or}}\,\,\,Q(S)=q(f(M))=\prod _{i=1}^{n}|f({\lambda }_{i})|,$$

where the *λ*_*i*_ denote the eigenvalues of *M*, and *f* is any well defined function (meaning well defined at those eigenvalues). Consequently any such *Q*(*S*) has a scaling function.

### Stochastic Growth and Scaling Laws

So far we have considered growth when a large network is developed by taking an increasing number of disjoint unions of isomorphic communities, and loosely tying them together. This approach was motivated by the high level of modularity that has been widely observed in real networks. The simplifying assumption that the communities are isomorphic allowed us to reach precise mathematical conclusions; but in practice we would expect to see modules of different types. In this section, we therefore test our conclusions on a more realistic, stochastic version of the model using real city data. This more general setting is not so amenable to analysis, and hence we give a computational experiment which shows that the same broad conclusions apply.

To set up the stochastic model, let *ψ* be a probability distribution defined over a finite subset  of , that is, those *S* for which $\psi (S) > 0$ is finite. Then suppose that we draw a sequence of graphs independently and at random from  using *ψ*, say {*S*_1_, *S*_2_, …}, and define the cumulative random graphsNow consider a performance measure *Q* defined over  and satisfying the conditions of Theorem 1. Then *Q* has a scaling law *G* such that (2) holds. In the stochastic setting of this section, let *q*_min_ = min{*Q*(*S*)|*S* ∈ } and *q*_max_ = max{*Q*(*S*)|*S* ∈ }. Under the reasonable assumption that *G*(*q*, *n*) is monotonically increasing with respect to *q*, it is clear that

$$G({q}_{{\rm{\min }}},n)\le Q(T(n))\le G({q}_{{\rm{\max }}},n),\,\,n > 1.$$

Hence networks with stochastic growth processes, defined over a finite set of generating networks, are sandwiched between scaling laws. We finish with an experiment on real data that tests the sharpness of these inequalities.

**Figure 1:**
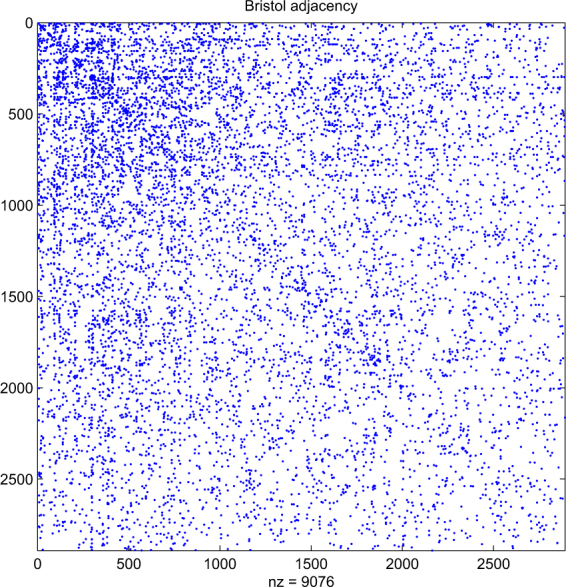
Adjacency matrix for Bristol network. Dots indicate nonzeros. Node ordering is arbitrary.

**Figure 2:**
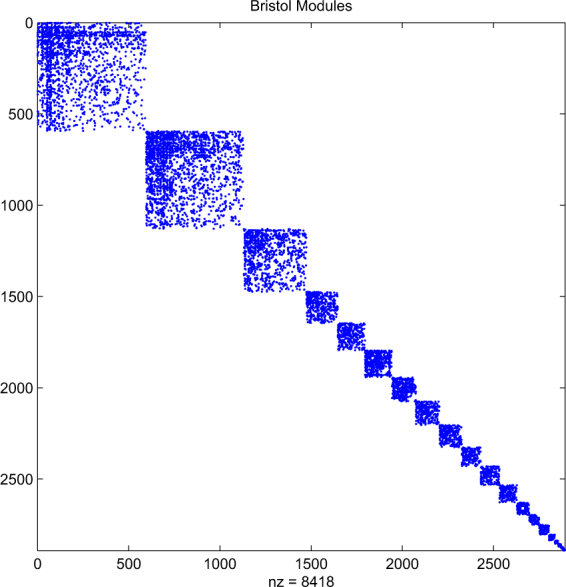
Modules for the Bristol network in Fig. 1.

For our computational test, we use a network from^6^ for the city of Bristol, UK. Figure 1 shows the adjacency matrix. Here, we have 2892 nodes and 4538 edges. Each node represents a Twitter account that has been geolocated to Bristol. An edge between two nodes indicates that the two accounts reciprocally mentioned each other at least once over the period 1–28 October, 2014. In^6^ this network was also broken down into 20 modules; these are shown in Fig. 2. This network has the same 2892 nodes, and contains 4209 edges; so the modules capture 93% of the original connections. The module sizes are 593, 536, 345, 172, 149, 147, 133, 129, 122, 104, 103, 95, 68, 54, 54, 32, 19, 16, 11, 10.

**Figure 3:**
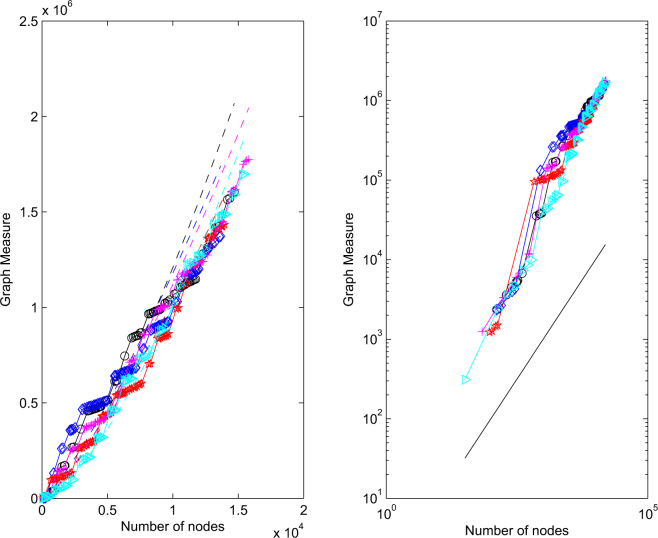
Five independent runs of network growth, showing network size against number of pairs of nodes connected by paths of length five or less.

**Figure 4:**
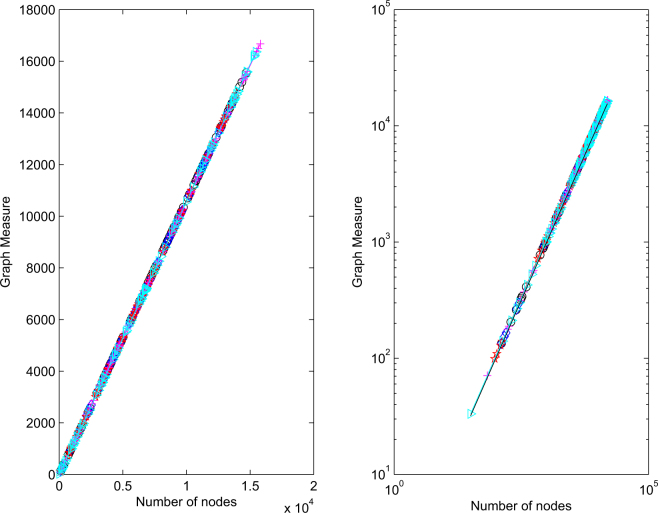
Five independent runs of network growth, showing network size against sum of Katz centrality values.

To produce Fig. 3, we repeated the same experiment over five independent runs. In each case, we built up a block diagonal matrix by drawing, with replacement, 100 modules. (So in the final network is about 5 times as big as Bristol. This is to show the asymptotic behavior as the network grows.) In other words, we built a block diagonal matrix, where the 100 diagonal blocks are drawn randomly from the collection of 20. For *k* = 1, 2, 3 …, 100 we then add three edges, each edge goes from a randomly chosen node in block *k* to a randomly chosen node in a different block. Hence, we add 300 new inter-block edges to connect up the network. Then we go through the final adjacency matrix and look at the subnetwork containing all the nodes in block 1, then all the nodes in blocks 1 and 2, then all the nodes in blocks 1, 2 and 3, and so on up to the final case of 100 blocks. So we are growing the network one block at a time, adding a small percentage of random edges. As our first network measure, we choose the number of pairs of nodes that are connected by a path of length five or less. The idea is that a short pathlength-based distance defines a plausible circle of people who might do favors such as find employment opportunities or provide some neighborly domestic support. We chose pathlength five because the individual modules are rich in pairs of nodes connected by shorter paths, and we wished to allow the extra random edges to have some effect. We note that this measure fits into the matrix function framework discussed above. The upper picture in Fig. 3 plots the number of such pairs against the network size. In the lower picture we repeat the same information on a log-log scale, with a reference line of slope 1 added. An underlying staircase effect can be seen, which is caused by the heterogeneity of the modules. A least-squares fit of a power law, log(*y*) = *α*log(*x*) + constant, for each run, produced values of *α* = 1.3945, 1.2483, 1.2893, 1.3161, 1.4605 over the five runs. Figure 4 gives the corresponding results for the case where the network measure is the sum of components in the Katz centrality vector. We recall^5^ that with an adjacency matrix *B*, the Katz centrality vector takes the form (*I* − *aB*)^−1^1. Here *a* is a parameter that must be chosen below the reciprocal of the spectral radius *ρ*(*B*). We note that *ρ*(*B*) is bounded by the maximum network degree. In our case the maximum observed degree was 54, so we used *a* = 1/60 throughout. Individual components in this type of centrality measure quantify the importance of network nodes, so the sum can be viewed as an indication of overall effectiveness of the network. In this case we see that the growth is very close to linear; least-squares power law values where 1.0044, 1.0023, 1.0015, 1.0024, 1.0043. Overall, the experiments are consistent with the results derived here, and with the many instances of empirically observed power-law like behavior in the experimental literature.

## Discussion

Our aim here was to develop simple, widely applicable, first principle arguments which show that power laws arise generically when some feature of a complex system is measured in terms of system size. The conclusions follow from high-level assumptions concerning the modularity of the underlying networks, the continuity of the network measure of interest and the existence of an underlying, possibly nonlinear, dynamical system describing its growth that has reached equilibrium. Further, the broad conclusions were shown to be robust in an experiment where a stochastic version of network growth was used. There are many ways in which these ideas could be pursued, including

- Customized analysis of specific network measures, in particular for the case where the Lipschitz-style condition (1) fails to hold,
- Rigorous analysis of the case of stochastic growth,
- Related analysis based on other models of network growth.

## References

[1] Newman MEJ (2012). Communities, modules and large-scale structure in networks. Nature Physics.

[2] Blondel, V. D., Guillaume, J.-L., Lambiotte, R. & Lefebvre, E. Fast unfolding of communities in large networks. *J. Stat. Mech*. P10008 (2008).

[3] Newman MEJ (2006). Modularity and community structure in networks. Proceedings of the National Academy of Sciences.

[4] Newman MEJ, Girvan M (2004). Finding and evaluating community structure in networks. Phys. Rev. E.

[5] Newman MEJ (2010). Networks: an Introduction.

[6] Grindrod, P. & Lee, T. E. Comparison of social structures within cities of very different sizes. *Royal Society Open Science* **3** (2016). 10.1098/rsos.150526 PMC4785974 26998323

[7] Decelle A, Krzakala F, Moore C, Zdeborová L (2011). Asymptotic analysis of the stochastic block model for modular networks and its algorithmic applications. Physical Review E.

[8] Clauset A, Shalizi CR, Newman MEJ (2009). Power-law distributions in empirical data. SIAM Review.

[9] Arbesman S, Kleinberg JM, Strogatz SH (2009). Superlinear scaling for innovation in cities. Phys. Rev. E.

[10] Bettencourt L, Lobo J, Strumsky D, West G (2010). Urban scaling and its deviations: Revealing the structure of wealth, innovation and crime across cities. PLoS ONE.

[11] Bettencourt L, West G (2010). A unified theory of urban living. Nature.

[12] Bettencourt LM, Lobo J, Strumsky D (2007). Invention in the city: Increasing returns to patenting as a scaling function of metropolitan size. Research Policy.

[13] DeLong J, Burger O (2015). Socio-economic instability and the scaling of energy use with population size. PLoS ONE.

[14] Leitão, J. C., Miotto, J. M., Gerlach, M. & Altmann, E. G. Is this scaling nonlinear? *Royal Society Open Science* **3** (2016). 10.1098/rsos.150649 PMC4968456 27493764

[15] Piovani D, Zachariadis V, Batty M (2017). Quantifying retail agglomeration using diverse spatial data. Scientific Reports.

[16] Schläpfer, M. *et al*. The scaling of human interactions with city size. *Journal of The Royal Society Interface* **11** (2014). 10.1098/rsif.2013.0789 PMC4233681 24990287

[17] van Raan AFJ, van der Meulen G, Goedhart W (2016). Urban scaling of cities in the Netherlands. PLoS ONE.

[18] Batty M (2013). The New Science of Cities.

[19] Bettencourt L, Lobo J, Helbing D, Kuhnert C, West GB (2007). Growth, innovation, scaling, and the pace of life in cities. Proceedings of the National Academy of Sciences.

[20] West G (2017). Scale: The Universal Laws of Life and Death in Organisms, Cities and Companies.

[21] Bettencourt LMA (2013). The origins of scaling in cities. Science.

[22] Higham DJ, Kalna G, Kibble M (2007). Spectral clustering and its use in bioinformatics. J. Computational and Applied Math..

[23] Higham NJ (2008). Functions of Matrices: Theory and Computation.

